# Whole Exome Sequencing of Growing and Non-Growing Cutaneous Neurofibromas from a Single Patient with Neurofibromatosis Type 1

**DOI:** 10.1371/journal.pone.0170348

**Published:** 2017-01-18

**Authors:** Daniel L. Faden, Saurabh Asthana, Tarik Tihan, Joseph DeRisi, Michel Kliot

**Affiliations:** 1 Department of Otolaryngology-Head and Neck Surgery, University of California San Francisco, San Francisco, California, United States of America; 2 Helen Diller Comprehensive Cancer Center, University of California San Francisco, San Francisco, California, United States of America; 3 Department of Pathology, University of California San Francisco, San Francisco, California, United States of America; 4 Department of Biochemistry and Biophysics, University of California San Francisco, San Francisco, California, United States of America; 5 Department of Neurosurgery, Northwestern University Feinberg School of Medicine, Chicago, Illinois, United States of America; Shanghai Jiao Tong University School of Medicine, CHINA

## Abstract

The growth behaviors of cutaneous neurofibromas in patients with Neurofibromatosis type 1 are highly variable. The role of the germline *NF1* mutation, somatic *NF1* mutation and mutations at modifying loci, are poorly understood. We performed whole exome sequencing of three growing and three non-growing neurofibromas from a single individual to assess the role of acquired somatic mutations in neurofibroma growth behavior. 1–11 mutations were identified in each sample, including two deleterious *NF1* mutations. No trends were present between the types of somatic mutations identified and growth behavior. Mutations in the HIPPO signaling pathway appeared to be overrepresented.

## Introduction

Neurofibromatosis type 1 (NF1), is an autosomal dominant disorder that affects approximately one in 3500 people[1]. The underlying cause is a heterozygous mutation in the Neurofibromatosis type 1 gene (*NF1*). Cutaneous neurofibromas (CN) are one the most frequent manifestations and a key portion of the diagnostic criteria. NF1 has considerable variability in clinical presentation among affected individuals, within families, and even within an individual throughout life[2]. CN can vary from a few to thousands, develop throughout life at different rates and may or may not continue to grow once they have appeared. The genomic underpinnings of CN growth and development are poorly understood.

Analysis of various types of tumors in NF1 patients, including CN, have demonstrated that independent *NF1* somatic mutations likely contribute to tumorigenesis in a “second hit” model[3]. Second hit mutations in *NF1*, when identifiable, appear at a distinct locus from the germline mutation, and from other somatic *NF1* mutations in separate tumors within the same individual, suggesting temporally distinct somatic events[4]. The type of mutation and genomic location, of both germline and somatic *NF1* mutations, likely effect CN burden and behavior. Limited genotype-phenotype correlations have been described related to the growth behaviors of CN in NF1 for both the specific germline *NF1* mutation and for acquired somatic mutations[5]. Numerous investigations have demonstrated that phenotypic expression of NF1 is likely significantly affected by the genotype at other modifying loci outside *NF1*, although the role of these mutations remains unclear[6]. For example, phenotypic similarity appears to decrease with the degree of relationship between individuals[7]. Traditionally it was believed that all CN harbored a somatic NF1 mutation. However, only about 60% of CNs have an identifiable *NF1* somatic mutation suggesting distinct somatic events outside *NF1* may be sufficient for tumorigenesis[4]. Some studies have suggested a role for microsatellite instability in NF1, although the specific effects on growth behaviors are unclear[7].

Whether the clinical phenotypic variability of CN growth patterns correlates with the germline *NF1* mutation type/location or the acquired somatic mutation type, location or timing, remains unknown. This is further complicated by the fact that over 1000 different mutations of the *NF1* gene have been identified across the spectrum of substitutions, indels, splice site alterations and gross chromosomal rearrangements, making the identification of causative associations difficult.

The fact that multiple CNs occur in a given individual provides an ideal, internally controlled, model for studying the isolated somatic events leading to CN formation and growth patterns. In order to investigate this relationship, we performed whole exome sequencing (WES) on six CN from a single patient and paired blood. Three of these tumors had demonstrated clinically measurable growth in size (growing tumors), while the other three had not changed in size during the same observation period (non-growing tumors).

## Methods

This project was approved by the IRB of UCSF and was given an exempt status an exempt status as all information was de-identified. Fresh-frozen tissue from surgically resected CNs and matched blood were obtained from a thirty-seven year old patient under Institutional Review Board approved protocols from University of California San Francisco. All tumor samples were snap-frozen at the time of surgery and stored at -80 until the time of processing. Six tumors from different body sites were selected for exome sequencing (3 growing, 3 non-growing) based on stringent quality assessment of normal and tumor DNA. Growing tumors demonstrated clinically apparent growth by serial physical exam and measurement by the senior author (MK) on 3–6 month serial exams, for greater than one year. Non-growing tumors were similarly evaluated. Before analysis, the diagnosis of each specimen underwent central pathological review and typical neurofibroma was confirmed. None of the tumors demonstrated atypical histology or evidence of malignant peripheral nerve sheath tumor. Snap frozen tumor tissue samples were analyzed by frozen section to assess neoplastic cellularity. Tumors were macrodissected to enhance tumor tissue, as confirmed by serial frozen sections. DNA was extracted using the QIAamp DNA Mini Kit (Qiagen, Hilden, Germany) according the manufacturers guidelines. Library preparation and whole exome sequencing was performed at Centrillion Technologies (Palo Alto, CA) using Agilent SureSelect Human All Exon v5 (Santa Clara, CA) on an Illumina HiSeq 2000 (San Diego, CA).

100bp paired-end reads were aligned against NCBI build 37 (hg19) of the human genome with BWA-MEM[8]. Duplicate reads were marked, local indel realignment performed, and base-quality scores recalibrated for each sample with the Picard suite (https://github.com/broadinstitute/picard) and the Genome Analysis Toolkit (GATK)[9]. Novel point mutations were identified using MuTect, while indels were identified using GATK Somatic Indel Detector, in tumor samples when compared against the normal[10]. Copy number segmentation was performed on log tumor/normal per-exon coverage ratios using CNVKit and the R package 'DNAcopy'[11,12]. Visual inspection of mapped reads within the entire *NF1* gene, and all identified mutations, was performed in IGV[13].

Pathway and network analyses were performed with Ingenuity Pathway Analysis (Qiagen, Redwood City, CA) by loading all high confidence mutations into the analysis platform with non-modified settings.

## Results

**Fig 1:**
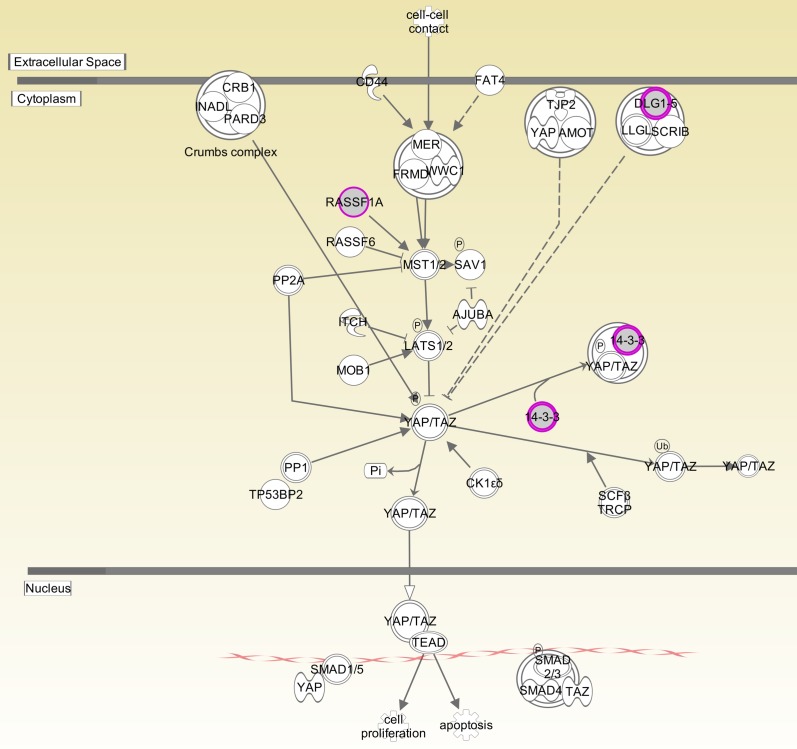
HIPPO Signaling Pathway. Components of HIPPO signaling pathway. Magenta circles represent genes/gene products mutated in NF1 tumors. 14-3-3 protein is encoded by *SFN* gene.

The mean depth of coverage across the seven samples was 40X-70X. Interrogation of the germline sample was performed and revealed a missense mutation in *NF1*, f2741v. Copy number analysis revealed no changes. Whole exome somatic mutation analysis identified 84 mutations total (15–26 mutations per sample) (S1 Table). After visualization in IGV this was narrowed to 32 high confidence mutations (1–11 mutations per sample). The average number of mutations per sample was five (Table 1). Two somatic mutations were found in *NF1*. Three mutations were cataloged in COSMIC (http://cancer.sanger.ac.uk/cosmic): *CD5*, *NF1* and *SFN*. Additionally, a mutation in *HLA-A* was present in the COSMIC Cancer Gene Census (http://cancer.sanger.ac.uk/census) as known to cause cancer. Canonical pathway overrepresentation analysis revealed the HIPPO pathway as significantly overrepresented (p value, 2.48E-4), which included the genes *SFN*, *RASSF1* and *DLG-4* (Fig 1). Biologic network overrepresentation analysis identified Nervous System Development and Function as the most significantly overrepresented (p value 4.98E-4), which included the genes: *DLG4*, *HFE*, *HLA-A*, and *NF1*.

**Table 1:** Somatic Mutations in Growing and Non-growing Neurofibromas.

		Samples						
		Growing 1	Growing 2	Growing 3	Non-Growing 1	Non-Growing 2	Non-Growing 3	Variant classification
	RASSF1	x						synonymous
	TRAV9-1	x						missense
	SUDS3	x						missense
	KLHL20	x						frameshift
	CD5	x						missense_variant
	DLG4	x						missense_variant
	WIBG	x						missense_variant
	UNC5CL	x						missense_variant
	HFE	x						missense_variant
	HECW1	x						missense_variant
	VPS36	x						synonymous
	COL22A1		x					synonymous
	UBASH3A			x				missense
	CYFIP2			x				missense
	PTPRZ1			x				sequence
	RYR1			x				synonymous
	NF1			x				stop gained
	NF1				x			disruptive inframe deletion
	DCAF13				x			missense
	PSD2				x			missense
	SLC25A14				x			synonymous, splice region
	NIPBL				x			missense
	ITIH1					x		missense
	INA					x		missense
	PRTG					x		missense
	TTC28					x		missense
	EIF4G1					x		missense, splice region
	ABCC3						x	synonymous
	SFN						x	synonymous
	CYP17A1						x	missense
	NOA1						x	missense
	HLA-A						x	missense

## Discussion

Patients with NF1 characteristically develop CN. The number, age of occurrence, and the growth rates of these tumors are highly variable among individuals and even within the same individual. The *NF1* gene encodes for the protein Neurofibromin. Neurofibromin is a negative regulator of the Ras/mitogen-activated protein kinase (MAPK) pathway[5]. As such, *NF1* is considered a classic tumor suppressor gene and its mechanism in NF1 is felt to be consistent with Knudson's two-hit hypothesis in which a patient carries a mutated germline *NF1* gene copy and tumor development, including CN, then requires a second hit[14]. While the germline mutations in *NF1* are well cataloged, with >1000 mutations identified to date, there is a paucity of information on the assumed acquired somatic mutations in CN[15]. This deficiency is likely related to both the difficulty of detecting somatic mutations in CN due to cellular heterogeneity within the tumors and also the small number of CN analyzed. Most analyses of somatic mutations in CN identify high confidence *NF1* mutations in only about half of the tumors[4,7].

While biallelic inactivation of *NF1* in CN is traditionally felt to be necessary for tumor development, somatic mutations affecting other tumor suppressor genes, (*TP53*, *CDKN2A* and *RB1)* have been identified in NF1 associated tumors including CN, suggesting that modifying loci may be sufficient for tumor growth in the absence of a second *NF1* mutation, or that the acquisition of a mutation at a modifying loci, in addition to a second *NF1* hit, may be a one of the factors affecting tumor growth behaviors[7]. Complicating this picture is the fact that the genotype-phenotype correlation of the underlying germline mutations is poorly understood and thus, make interpretation of all findings muddied. In order to control for this, we performed WES of three growing and three non-growing CN from a single patient with NF1 to investigate if similarities and/or differences in the types of somatic mutations between growing and now-growing CN could be identified.

It has been hypothesized that certain germline *NF1* mutations may predispose to particular phenotypes. For example, germline splice site mutations in *NF1* may increase the risk of tumor development[16]. Similarly, full *NF1* deletion is associated with early appearance and higher burden of CN[17], while a 3-bp in-frame deletion of Exon 17 is associated with fewer CN[18].

Therefore, as a first step, we interrogated the germline to identify the germline *NF1* mutation in this individual. A single missense mutation, f2741v was identified. While this mutation does not appear in COSMIC or cBioPortal (www.cbioportal.org), a frameshift insertion at the same location has been reported. Copy number analysis did not reveal any copy number variants.

Thirty-two high confidence somatic mutations were identified in the six samples. The only recurrently mutated gene was *NF1*, which contained two deleterious mutations: a stop gain (Growing 3) and an in frame deletion (Non-growing 1). Two other mutations in separate samples were also cataloged in COSMIC: *CD5* (Growing 1) and *SFN* (Non-growing 3). CD5 encodes a transmembrane glycoprotein that belongs to the highly conserved scavenger-receptor cysteine-rich superfamily. CD5 is felt to regulate antitumor immune response by potentiating tumor-specific T-cell reactivity[19]. The mutation at CD5 is a missense mutation, Ser485Cys, also reported in lung cancer (http://cancer.sanger.ac.uk/cosmic/mutation/overview?id=689345). The SFN (Stratifin) gene encodes for the 14-3-3σ protein. The 14-3-3σ protein regulates numerous cellular processes that are important in cancer biology, including apoptosis and cell-cycle checkpoints[20]. The mutation at SFN is a silent mutation, Cys96Cys, also reported in biliary tract cancer (http://cancer.sanger.ac.uk/cosmic/mutation/overview?id=5511476).

In addition to the mutations present in COSMIC, the gene HLA-A (major histocompatibility complex; class I; A) is present in the COSMIC Cancer Gene Census, which is a catalog of genes causally implicated in cancer. Loss of HLA class I antigens has been reported to be mechanism by which tumor cells escape immune attack[21]. The HLA-A mutation is a missense mutation, Lys292Glu. Four additional genes with mutations in our cohort have been reported to play a role in tumorigenesis in the literature: *TTC28*, *COL22A1*, *DLG4* and *RASSF1*. *TTC28* is a target of *TP53* and is reported to inhibit tumor cell growth[22]. *COL22A1* has been identified as a recurrently mutated gene in lung cancer[23]. *DLG4* has been suggested to function as a tumor suppressor and is involved in the development of HPV related cancers[24]. *RASSF1* is a recognized tumor suppressor. *RASSF1* promoter methylation is one of the most frequent alterations found in human tumors[25]. Lastly a missense mutation in *PRTG* was of interest as it has a known role in neurogenesis[26].

In order to investigate if any pathways were overrepresented in our gene sets we performed a canonical pathway overrepresentation analyses using Ingenuity Pathway Analysis. Interestingly, HIPPO signaling was identified as the single highly overrepresented pathway. The *SFN*, *RASSF1* and *DLG-5* gene products exist with this signaling cascade. HIPPO signaling is known to regulate cell cycle progression, apoptosis and cell differentiation[27]. Dysregulation of the HIPPO pathway is felt to contribute to cancer development through tumor initiation and progression and has considerable cross-talk with the WNT, SMAD and NOTCH pathways[27–29]. Dysregulation of the HIPPO signaling cascade has not previously been reported in NF1 and warrants further investigation.

One existing hypothesis regarding growth and arrest of CN is that *NF1* mutation triggers activation of Ras which then leads to oncogene induced senescence[30]. Additional mutations are then necessary for escape from this senescence, arguing in favor of modifying genes as a factor that could account for variable growth behaviors. This feedback cycle involves a number of genes as reviewed by Courtois-cox et al[30]. No mutations were identified in these senescence pathways. Further investigation of the transcriptome and epigenetic modifications of growing and non-growing CN may help further investigate this hypothesis.

While our study generates a number of interesting hypotheses for further investigation, there are a number of limitations. First, while our samples were separated into growing and non-growing by serial physical exam by a single author, a more objective measurement of growth was not obtained as the study was conducted in a retrospective fashion. The lack of an objective measurement of growth could account for a portion of the difficult in identifying a unifying theme among growing or non-growing CNs. Second, the fact that only 33% of the tumors had identifiable mutations in *NF1*, despite good coverage at the *NF1* locus, may reflect the known difficulty in identifying somatic *NF1* mutations due to cellular heterogeneity, or may reflect the concept that mutations at modifying loci may be sufficient for CN growth and account for differences in CN growth behavior. All samples appear to have a mutation in at least one gene that could potentially be causative; however, whether these mutations are passenger or drivers is unclear. There were no identifiable trends regarding the number of mutations or types of mutations within or between growing and non-growing samples as hypothesized. Additional studies with larger cohorts are needed to further investigate this question.

## Conclusions

CN growth behavior in NF1 is poorly understood due to the multitude of variables potentially effecting tumor development and growth. Here we performed WES on three growing and three non-growing CN from a single individual to test the hypothesis that somatic mutations in modifying loci could account for differences in growth behaviors between growing and non-growing CN. We identified between 1–11 mutations per samples with deleterious *NF1* mutations in two samples. While provocative mutations were identified in each of the samples at potential modifying loci, no trends were identified between mutations and in growing and non-growing samples. Mutations in genes in the HIPPO pathway appeared to be over-represented. Additional studies of the exome and transcriptome, as well as epigenetic modifications, in larger cohorts of growing and non-growing CN, are needed.

## Supporting Information

S1 Table Somatic mutations. All identified somatic mutations. Highlighted samples are high confidence.
(XLSB)
