# Correction: Whole Exome Sequencing of Growing and Non-Growing Cutaneous Neurofibromas from a Single Patient with Neurofibromatosis Type 1

**DOI:** 10.1371/journal.pone.0172620

**Published:** 2017-02-16

**Authors:** Daniel L. Faden, Saurabh Asthana, Tarik Tihan, Joseph DeRisi, Michel Kliot

The affiliation for the fifth author is incorrect. Michel Kliot is affiliated with: Department of Neurosurgery, Stanford University School of Medicine, Stanford, California, United States of America.
